# Analytical Method by Headspace‐Gas Chromatography for Determination of Six Residual Solvents in Losartan Potassium Raw Material

**DOI:** 10.1002/jssc.70319

**Published:** 2025-11-13

**Authors:** Vanessa B. de Camargo, Cássia V. Garcia

**Affiliations:** ^1^ Programa De Pós‐Graduação em Ciências Farmacêuticas (PPGCF), Faculdade de Farmácia Universidade Federal do Rio Grande Do Sul (UFRGS). Av. Ipiranga, Porto Alegre/RS Brazil

**Keywords:** headspace‐gas chromatography, losartan potassium, raw material, residual solvents, validation

## Abstract

Residual solvents, or organic volatile impurities, are a potential toxic risk for pharmaceutical products and have been a manufacturers’ concern for many years. Moreover, residual solvents can also affect the quality and stability of not only drug substances but also of drug products. The objective of this work is the development and validation of headspace gas chromatographic method for determination of six residual solvents (methanol, ethyl acetate, isopropyl alcohol, triethylamine, chloroform, and toluene) in losartan potassium raw material. Method development evaluated the critical parameters of sample diluent selection (dimethylsulfoxide and water), optimization of headspace conditions (incubation time and temperature), and chromatographic conditions (column temperature ramp speeds and sample split ratio). Validation was carried out in accordance to Brazilian guidelines. Dimethylsulfoxide was selected as the sample diluent, with an incubation time of 30 min at 100°C. The chromatographic determination was performed on a DB‐624 capillary column using programmed temperature, running time of 28 min and a split ratio of 1:5. The method proved to be selective, with suitable sensitivity (limits of quantification below 10% of the specification limits determined by the ICH), precise (relative standard deviations ≤ 10.0%), linear (*r* ≥ 0.999 for all solvents’ standard curves), accurate (average recoveries from 95.98% to 109.40%), and robust under the small modifications in the chromatographic conditions. A simple and reliable method was obtained. The analysis of a losartan potassium raw material batch has detected only isopropyl alcohol and triethylamine as residual solvents, indicating that purification processes applied to this active pharmaceutical ingredient production were capable to remove most solvents from synthesis step.

## 1 Introduction

The control of pharmaceutical impurities is a critical issue to the pharmaceutical industry. The presence of these unwanted chemicals even in small amounts may influence the efficacy and safety of the pharmaceutical products [1]. According to ICH guideline Q3A (R2) [2], impurities associated with APIs are classified into three categories: organic impurities (process and drug‐related), inorganic impurities, and residual solvents.

Residual solvents in pharmaceuticals (except for crude drugs and their preparations) are defined as organic volatile chemicals that are used or produced in the manufacture of drug substances or excipients, or in the preparation of drug products [3]. Organic solvents do not offer therapeutic benefits for patients, and they should be removed to meet the requirements of product specifications, good manufacturing practices (GMPs), and appropriate quality control [3, 4]. Many of the residual solvents have been proved to be severely toxic when consumed above the prescribed limits [5]. Also, their chemical identity and amount may affect some physicochemical properties of drug products, such as their particle size, crystalline structure, wettability, stability, and dissolution properties [6].

Gas chromatography (GC) is the preferred method for detecting residual solvents, and headspace (HS) sampling devices are the most used in pharmacopeias and in the literature [3, 4, 5, 6, 7, 8, 9, 10, 11, 12, 13, 14]. Some variations can be adopted, such as the use of low thermal mass (LTM) ovens to short the analysis time, through very high temperature program rates (up to 1800°C/min) [15] and the combination of GC with mass spectrometry (MS) for precise identification and quantification of contaminants [16]. It is also used for the analysis of solvents in other matrixes, such as intermediate products (spray dried dispersions) and food [17, 18].

Losartan [2‐butyl‐5‐chloro‐3‐[[4‐[2‐(1,2,3‐triaza‐4‐azanidacyclopenta‐2,5‐dien‐5‐yl)phenyl]phenyl]methyl]imidazol‐4‐yl]methanol), potassium salt, is a strong antihypertensive agent, non‐peptide, and exerts its action by specific blockade of angiotensin II receptors [19]. It can be produced by a variety of synthetic pathways and, in most of them, organic solvents are present in at least one step [20, 21]. According to ICH guideline [2], the solvents of the API synthesis pathway applied in this study can be classified as Class 2 (inherent toxicity—Methanol, Chloroform, Triethylamine, and Toluene) and 3 (less toxic—Isopropyl alcohol and Ethyl acetate). Even considering the clinical relevance of this drug in the last decades, there is no published work in databases focusing on losartan potassium residual solvents analysis up to now.

This work demonstrates the development and validation of a fast and sensitive analytical method by headspace‐gas chromatography (HSGC), using flame ionization detector (FID), to determine six residual solvents present in a synthetic pathway of losartan potassium raw material: methanol, isopropyl alcohol, ethyl acetate, chloroform, triethylamine, and toluene. It represents a great contribution to guarantee the safety and reliability of using this active pharmaceutical ingredient in the dosage forms production.

## 2 Experimental

### 2.1 Chemicals

Organic solvents dimethylsulfoxide (DMSO), methanol, isopropyl alcohol, ethyl acetate, chloroform, triethylamine, and toluene were purchased in GC purity grade from Scharlau, (Barcelona, Spain). The losartan potassium API was obtained from Vasudha Pharma Chem Limited (Hyderabad, India), declared purity of 99.6%.

### 2.2 Instruments and Analytical Conditions

An Agilent 7890A gas chromatograph equipped with FID detection, headspace sampler model 7697A, and software OpenLAB (Agilent Technologies, USA) was used. Separation was achieved on an Agilent DB‐624 column (30 m × 0.53 mm × 3 µm film thickness). Helium was used as the carrier gas with a constant flow rate of 4.718 mL/min (linear velocity of 34.104 cm/s). The oven temperature program was 40°C isothermal for 5 min, increased to 160°C at 10°C/min and then increased to 240°C at 30°C/min and held isothermal for 8 min. Sample was injected after an equilibration time of 30 min at 100°C. The syringe and transfer line temperatures were 105°C and 110°C, respectively. Split ratio was 1:5. The pressurization time was 1 min. The inlet and detector temperatures were 190°C and 260°C, respectively. The run time was 28 min.

### 2.3 Preparation of Standard and Samples

Standard solution of the solvents–methanol, isopropyl alcohol, ethyl acetate, chloroform, triethylamine, and toluene–was prepared from stock solutions of each one, diluted in DMSO GC grade, based on the ICH limits [2]. The final concentrations were: 600 µg/mL for methanol, 1000 µg/mL for isopropyl alcohol, 1000 µg/mL for ethyl acetate, 12 µg/mL for chloroform, 1000 µg/mL for triethylamine, and 178 µg/mL for toluene. A total of 5.0 mL of this solution was transferred to a 20 mL HS vial and capped and crimped immediately.

Sample solution of losartan was prepared by dissolving 200 mg of drug with 5.0 mL DMSO GC grade in a 20 mL HS vial. It was capped and crimped immediately.

All vials were taken to vortex shaker (IKA, Brazil) for 1 min.

### 2.4 Validation of Analytical Method

Validation parameters were evaluated according to RDC 166/2017 guideline (ANVISA, Brazil) [22].

**(a) Selectivity**

In order to demonstrate the method's capability to identify all target residual solvents in the API, the diluent (DMSO), standard solutions of individual solvents, mixture of solvents, API, and API spiked with mixture of solvents were analyzed under the proposed conditions.

**(b) Linearity**

This parameter was evaluated through three independent curves with six concentration levels of individual solvents standard solutions, in the range from LQ to 120% of the specified limit for each solvent.

Linear regression equations and correlation coefficients were calculated, as well as the statistical analysis using software Minitab 18.

**(c) Limit of Quantitation (LQ)**

To determine the LQ, solutions from individual solvents were prepared in decreasing concentrations and the signal to noise (S/N) ratio, that should be ≥ 10, was observed.

**(d) Repeatability and intermediate precision**

Six individual samples at the 100% level for each solvent were analyzed for repeatability. A second analyst carried out the analysis on a second day for intermediate precision.

RSD values were calculated for each condition.

**(e) Accuracy**

Accuracy was evaluated through recovery test by spiking known quantities of individual residual solvents in API samples, in three levels: low, middle, and high (in triplicate). The mixture of solvents and API sample solution were also analyzed.

**(f) Robustness**

Robustness was determined by small, deliberated modifications to the chromatographic conditions: oven initial temperature (± 5°C), gas linear velocity (29 or 39 cm/s) and column batch (batch USP445733H or USF226413H). RSD values were determined in comparison to nominal conditions.

### 2.5 Solutions Stability

The mixture of individual solvents solution and the sample solution spiked with residual solvents were kept under refrigerated temperature (2°C to 8°C) for 72 h and room temperature (25°C) for 24 h in HS vials. After the time intervals, they were analyzed using the proposed method. Values for RSD ≤ 20% were considered acceptable between initial and final amounts.

### 2.6 Determination of Residual Solvents in Losartan Potassium Batch

The validated headspace GC method was applied to determination of the residual solvents in a different batch of losartan potassium API.

## 3 Results and Discussion

Losartan potassium API was initially screened by GC procedure A from general chapter 467 of US Pharmacopoeia [11]. This general method demonstrated to be not adequate to quantify the solvent triethylamine, since the tailing factor did not fit the system suitability specification (< 2). In this way, the development of a new method was necessary.

One of the initial parameters evaluated during the method development were sample diluent (ultrapure water or DMSO). Water is the choice of the pharmacopoeial method [11] and DMSO an aprotic and polar solvent whose boiling point is higher (189°C) representing less interference in solvents analysis. Individual solvents dissolved in each diluent were analyzed to observe retention times and resolution between peaks. Results using DMSO showed more precision and sensitivity with higher recoveries.

The incubation temperature was also evaluated considering the boiling points from residual solvents to be determined and also from the diluents tested. In order to increase method sensitivity, low oven temperature requires higher equilibration times to reach good distribution between volatile compounds and diluents. When the HS equilibration temperature of 80°C with equilibration time of 60 min was evaluated, solvents with boiling points close to 100°C were not efficiently evaporated, resulting in low recoveries for solvents spiked in losartan potassium. When an equilibration time of 30 min was used along with temperature of 100°C, recoveries of the six solvents were satisfactory (between 80% and 120%). It was also observed that when temperatures of injection and transferred line were similar or 5°C higher than the HS oven (100°C) recoveries were not altered.

Considering chromatographic conditions, column selection is a crucial aspect. For this method, a 6% cyanopropyl/phenyl, 94% polydimethylsiloxane (DB‐624, Agilent) was selected due to its medium polarity, good resistance to higher temperatures and efficient separation between solvents with close boiling points. Most of Class 2 and 3 solvents listed in ICH Q3A [2] can be resolved by this column, except formic acid and acetic acid that are more polar.

Different column oven programs were tested (10–30, 10–40 e 10–50) for temperatures from 40°C to 240°C. Sample split ratios were also evaluated: 1:5 and 1:10. Another conditions applied were pre‐defined according to typical GC parameters for residual solvents [7, 8, 23, 24, 25, 26, 27]. The combined results indicated that the conditions established were the most adequate to an efficient and fast simultaneous determination of the solvents methanol, isopropyl alcohol, ethyl acetate, chloroform, triethylamine, and toluene in losartan potassium API.

### 3.1 Validation of Analytical Method

- **Selectivity**

**FIGURE 1:**
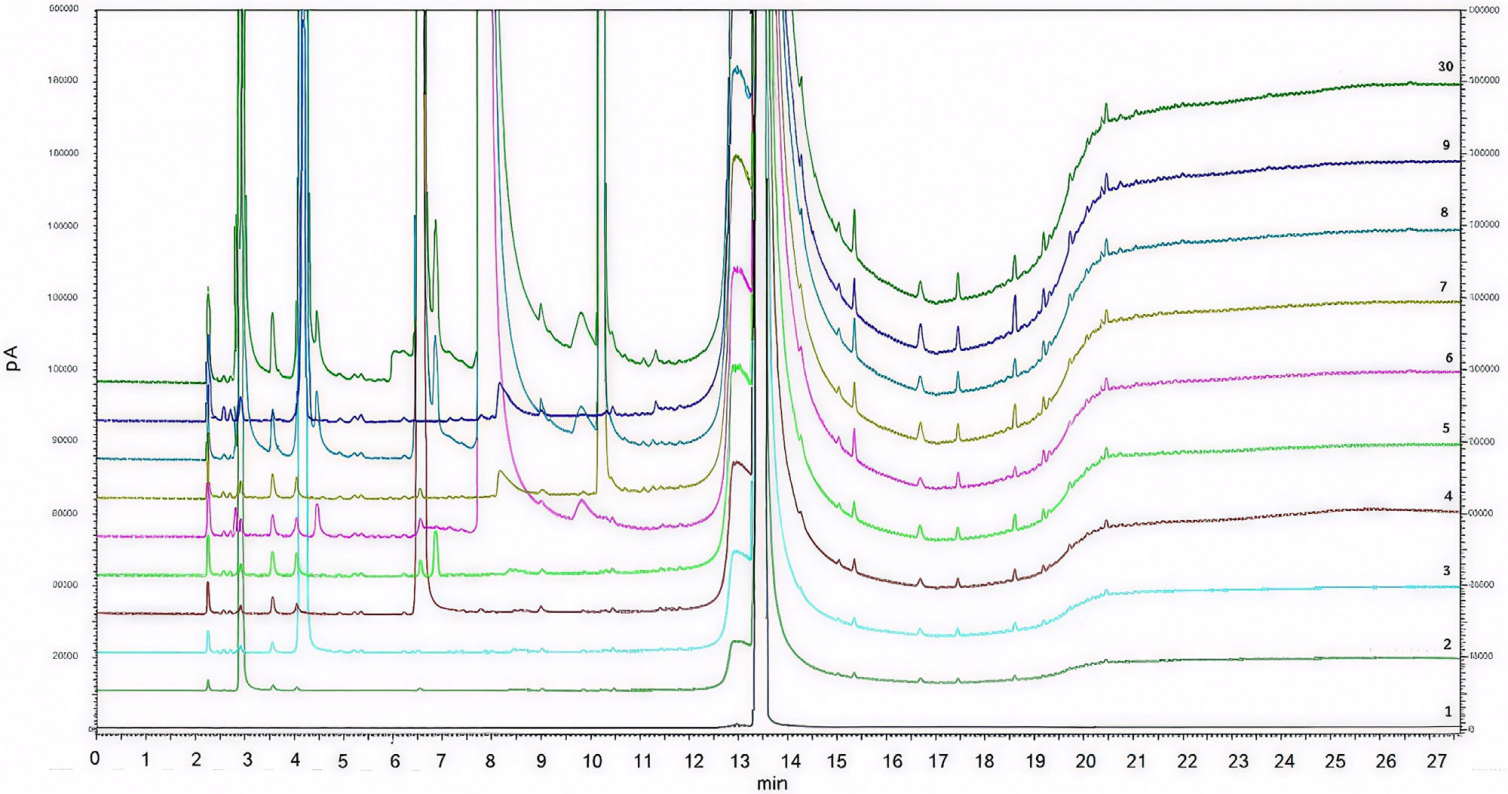
Overlap chromatograms of diluent DMSO (1), methanol (2), isopropyl alcohol (3), ethyl acetate (4), chloroform (5), triethylamine (6), toluene (7), mixture of solvents (8), API solution (9), and API solution spiked with mixture of solvents (10).

Figure 1 shows the overlap chromatograms for diluent (DMSO), standard solutions of individual solvents, mixture of solvents, losartan potassium (API), and API spiked with mixture of solvents. It is possible to verify the following retention times for the residual solvents peaks: 2.9 min for methanol; 4.2 min for isopropyl alcohol; 6.6 min for ethyl acetate; 6.9 for chloroform; 7.8 min for triethylamine, and 10.2 min for toluene. The resolution between ethyl acetate and chloroform was > 1.5. No interference from matrix was detected in the target solvents’ peaks, demonstrating the method's selectivity.

- **Linearity**

Table 1 demonstrates the results from linearity evaluation for each residual solvent by headspace GC method. Statistical evaluation was performed for each individual solvent regression data and all curves met the requirements for analysis of variance, residuals and outliers (data not shown).

- **LOQ**

**TABLE 1:** Results of validation parameters linearity, precision, and accuracy of headspace GC method for residual solvents determination in losartan potassium API.

Solvent	Linear range and regression equation (± SE)	Correlation coefficient (*r*)	RSD (%)	Recovery^a^ (%)
Methanol	3.0–720.0 µg/mL *y* = (15,090 ± 732)*x* + (1384 ± 63.5)	0.998	3.2 (*n* = 6) 4.0 (*n* = 12)	92.2 97.2 98.6
Isopropyl alcohol	3.5–1200.0 µg/mL *y* = (21,994 ± 202)*x*–(5781 ± 1,533)	0.997	3.1 (*n* = 6) 3.4 (*n* = 12)	110.4 108.5 109.3
Ethyl acetate	1.5–1200.0 µg/mL *y* = (67,361 ± 298)*x*–(5395 ± 512)	1.000	1.5 (*n* = 6) 1.5 (*n* = 12)	99.8 102.6 101.6
Chloroform	2.38–14.4 µg/mL *y* = (4,565.4 ± 22.6)*x*–(482 ± 210)	1.000	2.8 (*n* = 6) 2.4 (*n* = 12)	105.8 107.1 109.7
Triethylamine	1.0–1200.0 µg/mL y = (440,73 ± 2,672)*x* + (39,448 ± 7,224)	0.999	1.2 (*n* = 6) 1.1 (*n* = 12)	111.1 105.1 105.9
Toluene	0.89–213.6 µg/mL *y* = (91,355 ± 310)*x* + (1240 ± 1,375)	0.999	2.2 (*n* = 6) 2.2 (*n* = 12)	89.1 104.1 107.3

Abbreviation: SE, standard error.

^a^ Determined at three levels (low, middle, and high).

Results obtained from signal to noise ratio evaluation (≥ 10) were equivalent to 15 ppm for methanol (3.0 µg/mL), 17.5 ppm for isopropyl alcohol (3.50 µg/mL), 7.5 ppm for ethyl acetate (1.5 µg/mL), 12 ppm for chloroform (2.38 µg/mL), 5 ppm for triethylamine (1.0 µg/mL), and 4.45 ppm for toluene (0.89 µg/mL).

**FIGURE 2:**
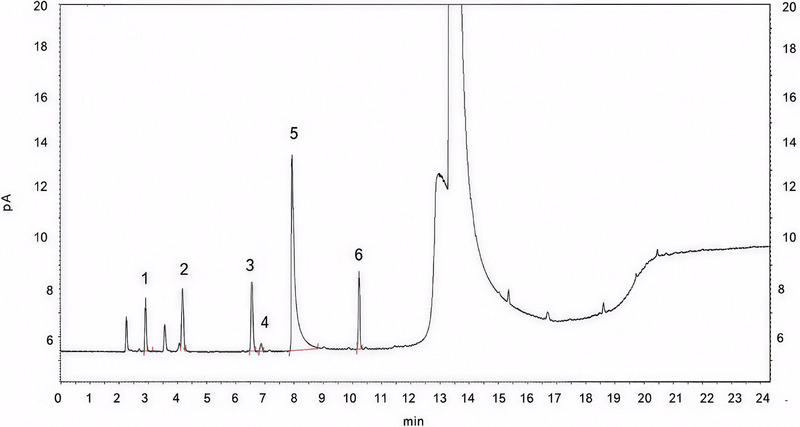
Chromatogram of mixture of solvents in their LOQ: (1) methanol, (2) isopropyl alcohol, (3) ethyl acetate, (4) chloroform, (5) triethylamine, (6) toluene.

A representative chromatogram of the mixture of solvents in their LOQ is shown in Figure 2.

- **Repeatability and intermediate precision**

Data from six solvents evaluated in 2 days by two different analysts showed RSD values less than 5%, which confirms the precision of the proposed method (Table 1).

- **Accuracy**

Results from recovery test indicate the GC method is accurate, since the recovery values obtained were between 80%–120% for all solvents in all levels tested (Table 1).

- **(f)Robustness**

As demonstrated in Table 2, the small modifications done in chromatographic conditions have not affected recoveries, which were very similar to those from nominal condition. System suitability parameters were not influenced by the modifications as well (data not shown).

**TABLE 2:** Results of robustness test for headspace GC method for residual solvents determination in losartan potassium API.

Solvent	Recovery (%) (RSD)					
	Nominal	Column batch USF226413H	Oven 35°C–240°C	Oven 45°C–240°C	Gas linear velocity 29 cm/s	Gas linear velocity 39 cm/s
Methanol	99.9	106.2 (4.3)	105.4 (3.8)	104.9 (3.4)	99.3 (0.5)	103.6 (2.5)
Isopropyl alcohol	103.1	109.3 (4.1)	108.1 (3.3)	107.3 (2.8)	102.2 (0.6)	107.3 (2.8)
Ethyl acetate	103.2	105.0 (1.2)	103.4 (0.1)	104.9 (1.2)	102.7 (0.4)	105.3 (1.4)
Chloroform	104.0	108.1 (2.7)	105.4 (0.9)	104.3 (0.2)	102.0 (1.4)	106.6 (1.7)
Triethylamine	102.5	102.5 (0.03)	101.6 (0.6)	102.6 (0.1)	100.8 (1.1)	101.9 (0.4)
Toluene	103.3	106.6 (2.2)	105.0 (1.1)	105.7 (1.6)	102.6 (0.5)	106.1 (1.8)

### 3.2 Solution Stability

Results of stability study of solutions are presented in Table 3.

**TABLE 3:** Results of stability study of mixture of solvents solution and sample spiked with solvent solution (control).

	Mixture of solvents (recovery %) (RSD)			Control sample (recovery %) (RSD)	
Solvent	Fresh solution	Room temperature 24 h	Refrigerated 24 h	Fresh solution	Room temperature 24 h
Methanol	99.2	110.8 (7.8)	175.7 (39.3)	105.2	105.7 (0.02)
Isopropyl alcohol	99.0	113.3 (9.6)	180.9 (41.4)	108.4	112.8 (2.8)
Ethyl acetate	99.7	98.9(0.6)	163.8 (34.4)	104.5	103.0 (1.02)
Chloroform	99.3	105.9 (4.6)	166.8 (35.9)	107.8	104.3 (2.3)
Triethylamine	101.4	99.9 (1.0)	142.9 (24.0)	104.1	100.6 (2.5)
Toluene	100.9	99.6 (4.3)	157.6 (27.8)	105.9	100.2 (3.9)

For the mixture of solvents solution and sample spiked with solvents solution, it was not observed differences in recoveries, and all RSD values were below 20% for methanol, isopropyl alcohol, ethyl acetate, chloroform, triethylamine, and toluene under room temperature condition. According to chapter 1467 from US Pharmacopoeia [11], the recommended recovery is between 80%–120%. However, for those maintained under refrigeration, mixture of solvents solution demonstrated recoveries above the expected and high RSD values, which indicates this condition is not adequate to store the solutions and should be avoided.

### 3.3 Determination of Residual Solvents in Losartan Potassium Batch

**FIGURE 3:**
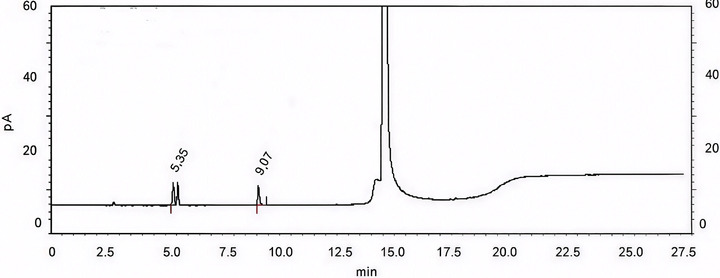
Chromatogram of losartan potassium API batch obtained through the headspace GC proposed method for residual solvents (Rt = 5.35 corresponds to isopropyl alcohol and Rt = 9.07 corresponds to triethylamine).

The developed and validated method was applied to a different batch of losartan potassium API. Although six solvents were present in the synthesis pathway of this API, only isopropyl alcohol and triethylamine were detected in the batch. For the first, a low toxic potential solvent (Class 3) [2], the concentration found, 49 ppm, corresponds to 1% of specification limit (5000 ppm). For the latter, the concentration found was below the LOQ of the method 5 ppm (1.0 µg/mL of triethylamine). A representative chromatogram is shown in Figure 3.

The analysis of the API batch demonstrated that purification processes applied to this API production were capable to remove most solvents from synthesis step.

Analytical methods focusing on the determination of residual solvents in losartan potassium API were not found until now. Solvents determined in the proposed headspace GC method can be detected and quantified at levels less than 10% of the limits from ICH Q3A guideline [2]. Besides, incubation times and running time were lower than those from the US pharmacopoeia general method [11], which was the first one to be considered.

## 4 Conclusions

The proposed headspace GC method for determination of residual solvents methanol, isopropyl alcohol, ethyl acetate, chloroform, triethylamine, and toluene in losartan potassium raw material was successfully developed and validated. It can be adopted in the quality control of this drug, even if the residual solvents are in low concentration, due to its sensitivity at levels less than 10% of the limits from the ICH Q3A guideline and accuracy. Low incubation and running times also contribute to fast routine analysis in pharmaceutical industries.

## Author Contributions

**Vanessa B. de Camargo**: investigation, methodology, validation. **Cássia V. Garcia**: supervision, roles/writing – original draft, writing – review and editing.

## Conflicts of Interest

The authors declare no conflicts of interest.
